# Perspectives on substance use among youth with chronic medical conditions and implications for clinical guidance and prevention: A qualitative study

**DOI:** 10.1371/journal.pone.0209963

**Published:** 2019-01-23

**Authors:** Elissa R. Weitzman, Parissa K. Salimian, Lily Rabinow, Sharon Levy

**Affiliations:** 1 Division of Adolescent/Young Adult Medicine, Boston Children’s Hospital, Boston, Massachusetts, United States of America; 2 Department of Pediatrics, Harvard Medical School, Boston, Massachusetts, United States of America; 3 Division of Developmental Medicine, Boston Children’s Hospital, Boston, Massachusetts, United States of America; University of Queensland, AUSTRALIA

## Abstract

Increasing numbers of youth globally live with a chronic illness. These youth use alcohol and marijuana at levels equal to or greater than their healthy peers and, when using, are at elevated risk for regular or problem use and adverse consequences to their condition. Little is known about whether behavioral theories commonly invoked to explain adolescent substance use apply to this group, limiting our ability to develop, tailor and target preventive interventions. We interviewed youth ages 16–19 years in care for a chronic disease to gain knowledge of this group’s perspectives on substance use risk, decision-making, and preferences for clinical guidance. Interviews were transcribed and thematically analyzed. Three principal themes emerged: first, having a chronic disease frames understanding of and commitment to health protecting behaviors and impacts decisions to avoid behaviors that carry risks for disease complications and flares; second, developmental impulses typical of adolescence can amplify an adolescent’s propensity to take risks despite medical vulnerability and direct youth toward maladaptive choices to mitigate risk; and third, poor knowledge about effects of substance use on specific features of a disease shapes perceived risk and undermines health protecting decisions. Youth navigate these issues variously including by avoiding substance use at a specific time or entirely, using while cognitively discounting risks and/or adjusting treatment outside of medical advice. Their perceptions about substance use are complex and reveal tension among choices reflecting a chronic illness frame, developmental impulses, and knowledge gaps. Delivery of targeted guidance in healthcare settings may help youth navigate this complexity and connect patient-centered goals to optimize health with health protecting behavioral decisions.

## Introduction

Today’s population includes an unprecedented number of adolescents–some 1.8 billion globally [1]; 22.9% of the US population is less than 18 years of age [2]. While adolescents are generally considered a healthy group, in the US one-of-four is growing up with a chronic disease as are sizeable percentages of youth elsewhere [3]. Youth affected by chronic illness experience significant levels of disease and treatment burden (e.g., pain and medication side effects), which undermine wellbeing, lead to frequent and costly healthcare utilization [4] and family financial problems [4]. By adulthood, chronically ill youth face outsize risks for poor educational, relationship, economic and health outcomes [5,6]. Lifecourse risks reflect the complex interplay of disease and treatment experiences and the cumulative effects of social isolation, victimization, school disruption, psychological injury and home life strain [7]. Substance use poses a unique and modifiable health risk for these youth that may worsen a myriad of life outcomes, underscoring the importance of its prevention.

Alcohol and marijuana use often begins and escalates, in adolescence [8,9]. These behaviors are influenced by many factors including a natural drive towards rewarding activities [10], the availability of substances [11], modeling of peer behaviors [12,13], and perceptions of harm and benefit [14–16]. Health behavior theories centered on cognitive and social processes encapsulate these factors [17] and have been used to inform substance use preventive interventions [13]. For the many US youth with a chronic medical condition (YCMC) [3], and the growing percentage of youth globally with a chronic disease [18–25], decisions regarding substance use may be further influenced by their knowledge and concerns about the effects of substances on their health overall, their condition specifically and its treatment.

Research with US samples indicates that substance use is prevalent among YCMC: approximately one-third drink alcohol in high school of which one-third do so at a binge level; one-fifth use marijuana [26]. Compared to their healthy peers, YCMC are as likely to initiate alcohol use and more likely to initiate tobacco and marijuana use in early adolescence (ages 14 or younger); once using these substances, they are more likely to progress to regular or heavy/problem use by late adolescence/young adulthood [26,27]. For *all* youth, substance use poses risks for acute harm from accident and injury [28]. For YCMC, substance use poses additional risks from adverse medication interactions [29], increased treatment non-adherence [26], and poor disease control [30]. Indeed awareness of these factors contributes to reduced alcohol use among youth taking medications that have alcohol use contraindications.[31] To date however, few studies have considered how insights from behavioral theories stipulating important sources of influence on substance use might apply to medically vulnerable youth specifically. Gaps limit our ability to develop, tailor, and target to YCMC substance use-related preventive interventions and clinical guidance.

Prevention efforts grounded in communicating information about health risks related to substance use are common [32–35]. They emphasize perceived susceptibility to a health threat, seriousness or severity of the threat, and appraisal that steps to reduce the threat are achievable and beneficial (consistent with dimensions of Health Belief and Social Cognitive Models) [32,35,36]. Generally speaking, interventions that center *solely* on these factors have limited utility for preventing adolescent substance use [37]–perhaps due in part to neurodevelopmental patterns affecting judgment and decision-making. Brain maturation in early adolescence occurs in regions of the brain that are tuned to exploration, sensation seeking and sociability; maturation in early adulthood occurs in regions that govern deliberative reflection and are best suited to considering risks [10,38,39]. This normal developmental progression may leave adolescents susceptible to the appeal of exciting and social stimuli at a time during which they are less able to reflect on and revise behaviors to avoid harm.

Despite this, it is possible that information about specific health risks might be valued by and impactful for youth with a chronic disease. These youth have firsthand experience with serious health harms and may experience a heightened sense of susceptibility with strong motivation to avoid risks, potentially boosting the value of well targeted guidance. While behavioral theories that emphasize social norms and peer influence on substance use behaviors [40,41] might also be relevant, they too may imperfectly apply to medically vulnerable youth who may not fully identify with the norms, models and activities of healthy peers. Similarly theories that emphasize the role of supply-side factors including advertising and promotion of substances [42] may poorly explain YCMC substance use if priorities to remain symptom free, in control, clear-headed and attuned to a disease state override the appeal of market-based patterns. These issues are not straightforward since youth with a chronic illness may perceive that substance use confers condition-related benefits and a means to escape having a disease, relieve anxiety, and in the case of marijuana address symptoms or treatment side effects.

In light of these complexities and the clear need to identify leverage points to delay and reduce substance use among youth with chronic illness, we undertook a qualitative study of beliefs and attitudes toward substance use among adolescents growing up with a chronic illness. We sought to understand factors that contribute to YCMC decision-making around substance use and to elicit preferences for the content and delivery of clinical guidance that could contribute toward risk-reducing and health promoting decisions by tapping into concerns related to patient-centered harms and outcomes.

## Methods

### Participant selection

Participants were recruited from an existing cohort of youth enrolled in a clinic-based validation study of a brief screening tool for assessing alcohol use risk among YCMC [26]. Details of the parent study are described elsewhere [26]. All youth had been diagnosed for at least one year. For this qualitative study, purposive sampling was used to select and interview a subgroup of eligible adolescents ages 16 to 19 years that represented a mix of gender, disease type, and history of substance use. Participants who opted out of research re-contact in the parent study or were unable to participate in a private narrative interview were ineligible. Participants were mailed a $25 gift card as compensation for their participation. The Boston Children’s Hospital Institutional Review Board approved the study with a waiver of parental consent for youth participants under the age of 18 years. All participants provided age-appropriate verbal consent/assent.

### Interviews

Telephone interviews were conducted in English by one or two researchers (PKS and LR) calling from a private office and lasted between 30 and 90 minutes. Interviewers were both female, had been trained in qualitative research interviewing using non-directive language, had one to two years of prior research experience recruiting similarly aged adolescents for clinical epidemiologic studies. Interviews were conducted by telephone for convenience and to reduce barriers to participation for adolescents, who can be a hard to reach group, and given the potential that eligible participants might live at some distance from the hospital and research site. The research team did not have clinical relationships with study subjects. Interviews were conducted at a time requested by participants, to optimize convenience and privacy and to limit burden. To participate in the interviews, participants were asked whether they were in a private place where they would not be overheard–all participants verbally affirmed this. Interviews were audio-recorded with participant permission and transcribed verbatim.

Interviews followed a semi-structured guide (see Supplemental Information) developed to explore daily experiences of the patient’s medical condition, and to elicit information about personal, social, situational, and clinical factors affecting decision-making around alcohol and marijuana use. Preferences for clinical communication about substance use were also elicited. Questions were grounded in social cognitive theories of adolescent risk taking and focused on perceived risk of substance use [15,43–45] and the salience of social influences on health behaviors [16,46–48]. Interviews were undertaken in three waves over the course of one year to enable the study team to refine the question guide in response to emerging findings, and to afford opportunity to expand upon and validate themes consistent with pragmatic goals of balancing codes developed using deductive (theory-based or *a priori*) and inductive (emergent) approaches[49][50][51][52].

### Data analysis

All interviews were transcribed and texts were then analyzed using an iterative, process that included within and across transcript coding to identify emergent themes, consistent with prior work [53,54] following a grounded theory approach[55,56]. Two analysts (PKS and LR) collaboratively generated an initial coding framework, informed by theory [57]. This framework evolved during transcript review from annotation of major themes using open coding (i.e., the chronic illness experience) to articulation of subthemes and constructs using axial and selective coding. Each analyst independently read and coded each transcript, labeling and organizing themes into notes for joint review and discussion. As refinement of the coding scheme progressed, principal and subordinate themes were specified that reflected areas of consistent commonality across the sample, i.e., ideas that were reflected in responses from the large majority or all participants. Data were organized into major and minor or subordinate themes and data tables were developed populated with quotes representing comments from all of the study participants. The process was iterative, and reflected both deductive and inductive processes: development of initial interview questions drew on principles of existing social behavioral theories (a deductive process) while content coding of themes/observations and stories shared by participants was undertaken to arrive at areas of meaning for this sample (an inductive process). Multiple rounds of selective coding were undertaken by the team to group themes and subthemes into a coherent model. Finally the set of exemplary quotes used in the data tables was winnowed and reduced for final reporting. Study principal investigators (ERW and SL) reviewed the coding scheme, themes and tables for coherence, consistency, and correspondence with transcripts. Interviewing continued until thematic saturation was reached (achieved for 31 youth) under a consensus-driven process. Methods and reporting align with comprehensive standards for qualitative research [58].

## Results

### Sample characteristics

In total, 36 youth consented to participate of 39 who were invited (92.3% consent rate). Thirty-one youth completed an interview (participation rate 79.5%, non-completion was due to youth scheduling difficulties). Six interviews conducted with patients with attention-deficit/hyperactivity disorder (ADHD) were excluded from the final sample due to the fundamental differences between neurodevelopmental conditions (i.e., ADHD) and the physical conditions affecting the majority of the sample. The final sample comprised 25 youth (14 female) aged 16–19 years (mean age = 18.0): 11 with a rheumatic disease, including two with inflammatory bowel disease (IBD)-associated arthritis and eight with juvenile idiopathic arthritis (JIA), and one with polymyositis; four with IBD only; nine with Type 1 diabetes (T1D); and one with moderate persistent asthma. The sample was predominantly white, and most participants reported at least one parent with a college education living with them at home (see Table 1). The average age of diagnosis was eight years, and on average, participants visited the subspecialty care clinic where they were originally enrolled 3.4 times in the year preceding enrollment into the parent study.

**Table 1:** Characteristics of participants.

Characteristics^a^	No. (%)
Total	25
Average age in years, (SD)	18.0 (1.12)
Sex	
Male	11 (44.0)
Female	14 (56.0)
Race^b^	
White	19 (79.2)
Other	5 (20.8)
Highest level of education of parent(s) living at home^b^	
Graduated college or more	16 (66.7)
Less than college	8 (33.3)
Diagnosis	
Type 1 diabetes	9 (36.0)
Juvenile idiopathic arthritis	8 (32.0)
IBD-associated arthritis	2 (8.0)
Other rheumatic disease	1 (4.0)
IBD only	4 (16.0)
Chronic persistent asthma	1 (4.0)
Average age in years at diagnosis, (SD)	8 (4.15)
Average number of subspecialty clinic visits in the past year, (SD)	3.44 (1.76)

Abbreviation: IBD, inflammatory bowel disease.

^a^ Values are the number (percentage) unless indicated otherwise.

^b^ Values may not equal total due to missing data.

### Principal themes

Three principal themes emerged around substance use decision-making. The first theme, the chronic disease frame, describes ways in which living with and managing a chronic condition shapes how affected youth view and approach the world and within that, understand and commit to health behaviors. The second theme, the adolescent frame, pertains to the influence on substance use decision-making of developmental status, attendant impulses toward novelty and experimentation, and sensitivity to social influences. The third theme, disease-specific substance use knowledge, encompasses understanding of the connections between substance use and a chronic condition as they may influence perceived risk and vulnerability, and within that preferences for content and delivery of substance-use related education and guidance.

### Theme 1: The chronic disease frame

For participating youth, living with and managing their chronic condition shapes the way they define self and experience life. This chronic disease frame pervades decision-making about health behaviors. Youth reported a strong sense of health consciousness centered on avoiding disease complications. Substance use decisions were filtered through this lens. Consideration of health and well-being motivated youth to abstain from or moderate substance use or, in some cases, prompted use for instrumental purposes, such as to relieve pain or symptoms (Table 2). Subthemes are outlined below.

**Table 2:** Theme 1. The chronic disease frame.

Subtheme	Concept	Exemplary quotes
Impact of condition on daily life and identity	Condition as core feature of identity	…I’ve only really lived life with [T1D]…so I don’t really know what I’m missing. (18-year-old male with T1D) …[Diabetes is] a huge part of my life. I wake up every morning, I test my blood sugar. Like depending on that I’m either feeling awful or great if it’s high or low or perfect…my whole life kind of revolves around trying to keep it as good as I can. (19-year-old male with T1D) I mean I grew up wanting to do different stuff but just never being able to do it [because of my arthritis]. Like I wanted to do gymnastics when I was little. But you know like I think if I didn't have arthritis I'd be doing different things. But I grew up with arthritis so I don't really know. (17-year-old female with JIA)
	Illness intrusiveness	…I've missed a lot of days of school in the past. Like, I've missed 60 days because of arthritis. It was a little hectic. (16-year-old male with JIA) When I’m not feeling well or I’m stuck in the hospital, I’m not really going to get a chance to change my outlook on life. (17-year-old male with IBD) A lot of the time I didn't feel like necessarily going out to do anything [because of my health] …if I just really wasn't feeling up to it, I'd tell [my friends] "I can't" or "I'm feeling really sick."…I kind of felt like maybe people were doubting whether or not I was actually sick all the time. (17-year-old male with IBD)
	Conceptualizing healthy as being symptom and complication free	Being healthy for [my friends] is just exercising and eating healthy. Whereas for me it’s having the full energy to do stuff and make sure there are no flares. (19-year-old female with IBD) To me I guess [being healthy] would be just being able to …kinda just do your everyday life without something getting in the way all the time. (19-year-old female with JIA) I think healthy is kind of like when your body is working the way it should. Like you're not sick, you can do whatever you need to do in a day without an issue. (18-year-old male with T1D)
	Coming to terms with having disease	You just have to realize that things can get better but you have to take all the medications that are prescribed to you …I think I just realized like how severe the illness was. (19-year-old female with IBD-associated arthritis) …I had a decision to make: that I could let diabetes run my life or I could run my life. (18-year-old female with T1D)
	Accelerated or interrupted maturity	[I’m] probably honestly more …mature [than my peers]. I think because I had to grow up at such a young age. And I find myself not finding the same things funny and not really into the whole partying…it’s almost like I just skipped a couple years. (19-year-old female with JIA) I had to mature a little faster [than my friends]…I mean, [Crohn’s is] kind of an adult thing to have to deal with. (19-year-old female with IBD) When Crohn's first hit me, it was kind of during a time when I was becoming a teen, so I didn't really mature as much then. But once I wasn't affected by the Crohn's so much, then I kind of went through it faster. (17-year-old male with Crohn’s)
Health-conscious influences on substance use decisions	Consideration of consequences on chronic condition	…I always thought about how [drinking] would affect my Crohn’s. I’ve always heard about like alcohol can disturb the stomach lining. Therefore I was like, well, if my stomach’s already affected, why add poison to it? (19-year-old male with IBD) I think because [managing my arthritis] consumes so much of my life that I wouldn’t want to just like throw it away in one night [of drinking]. (19-year-old female with JIA) If you drink way too much and you don’t give yourself enough insulin, you wake up and your blood sugar is way too high and you feel like crap. (17-year-old male with T1D) When you are drunk…God forbid if I get sick enough that I’m like throwing up, that’s like always scary because you never know what’s going to trigger this disease because there isn’t concrete stuff on it in terms of food or whatever. (19-year-old female with IBD)
	Consideration of alcohol-medication interaction consequences	I know I can't really skip [my medication] because then my knees are going to hurt really bad. So I guess if I ever drink in the future, I'll just barely have any because I'm afraid that it's going to like cancel it out or something. (16-year-old female with JIA) When I was diagnosed like I felt really sick, like couldn't walk or anything. If I do [drink alcohol] and the medicines stop working, I don't want to go back to where I was. I'd rather just stick with the medicine and have it work. (16-year-old female with polymyositis) Other people can just get black-out drunk and they're fine the next day, but I know I could absolutely never do that [because of my medication]. And it's hard because you do those kind of things to like, relieve stress, but those kind of things actually stress me out more because I know that like, if I'm doing it, it's always risky [because I’m taking methotrexate]. (18-year-old female with JIA)
	Conditioning abstinence on perception of disease activity	If one day I’m planning on going out with my friends and my stomach hurts…I won’t drink…I always listen to my body. (19-year-old female with IBD) Well I [decided not to drink that night because I] was just listening to my body, I guess. I didn't feel great. You know, when my blood sugar’s pretty high like that I just don't feel good at all. I feel like, dehydrated, and I just feel kind of sick. So you know once I start feeling like that, I really try to focus first on getting my blood sugar in check so I can feel better first and then go back to whatever I was doing. (19-year-old male with T1D)
	Valuing clear-headedness to accurately perceive feedback about disease activity and symptoms	It’s just all about feeling if you’re high or low. And you can lose that if you get too drunk. (18-year-old male with T1D) So when people drink alcohol, I don't think they are going to pay attention to their symptoms. Because the brain is not in place to pay attention to what they are doing …I like to have the knowledge of what I'm doing. (17-year-old male with T1D) [When I smoked marijuana] I thought I couldn’t breathe. [My friends] weren't sure what to do because they weren’t sure if it was just because I had you know, had [marijuana] in my system, or like was it because I'm actually hurt and slowly not being able to breathe. (19-year-old female with asthma) When you are drunk, you don't feel things as strongly as like other times …because those feelings are kind of lessened, I’m not as able to be like, “well this is feeling…” you know something like that. (19-year-old female with IBD) Yeah, [I’ve given] a lot of thought [to how my body might react when drinking], actually ‘cause like you can’t trust other people to know what’s happening to your body so you have to be able to tell for yourself. (17-year-old female with T1D)
	Using substances for symptom relief	[Marijuana] like *killed* my Crohn’s pain…I noticed that out of all the pills I did, me smoking marijuana in a five-year span, I’ve had far less hospitalizations, far less attacks, so I pretty much just stuck with it. (19-year-old male with IBD) …I smoked because it would make my knees stop hurting or help me sleep because I usually can’t sleep at night. (16-year-old-female with JIA) …when you have arthritis, like everything is so tight…and when you drink it’s just sort of like you’re more loose. (18-year-old female with JIA) If I know I'm feeling like my stomach's like upset and I'm having like loose stool and whatnot it’s like, oh, well maybe if I try to smoke maybe this will help. (19-year-old female with IBD-associated arthritis)

#### Impact of condition on daily life and identity

Having a chronic condition was inextricably tied with everyday life, and for some youth constituted a stable feature of self and identity. Having a chronic condition increased some youth’s sense of inner strength (hardiness), conferring empathy and perspective; others had not fully reconciled their condition as a permanent feature of life and self. Youth described this coming to terms with their condition as a process that included accepting responsibility for disease management and becoming motivated to preserve health. Many participants reported feeling a sense of accelerated maturity and greater life experience relative to their peers which stemmed from having to manage their condition. Where present, this diminished their interest in heavy and in some cases any substance use. Others felt that time spent away from their peers for health reasons interrupted their social maturation; in rare cases, youth viewed substance use a means of catching up and behaving in an age or developmental stage appropriate fashion.

Daily disease experience and adjustment to having a chronic condition were integrally connected to health-related decision-making. Youth reported high value for minimizing illness intrusiveness and disease-related hassles–whether short-term interruptions (e.g., missing class for “15 minutes here, 15 minutes there” to check blood sugar, as shared by a participant with Type 1 diabetes), or larger dislocations (e.g., “having to drop out of school and get my GED (general education diploma) [due to arthritis flares],” an example given by a participant with JIA). Being healthy was highly motivating and conceptualized as living complication-free and unimpeded in activities.

#### Health-conscious influences on substance use decisions

Consciousness about health status and vulnerability played a central role in participants’ decisions around substance use. When discussing their decision-making process, youth reflected on how drinking may complicate their condition. Many cited concerns about the interaction of alcohol with their medications. Others worried about short-term consequences, such as acute hypoglycemia for youth with type 1 diabetes. The presence or absence of physical symptoms weighed into this group’s immediate decision-making, and many youth reported deferring substance use/abstaining when symptomatic. Participants were acutely aware of the need to stay physiologically and cognitively attuned to their symptoms, and worried that being under the influence could cloud perception of somatic information necessary to staying aware of their disease state. The perception that using substances may ameliorate symptoms also factored into participants’ decisions to use substances. Some youth reported using marijuana for pain relief or for “stomach calming” effects in the case of IBD.

### Theme 2: The adolescent stage

When discussing their substance use decisions, youth cited motivations and influences typical of their developmental status–these included valuing novelty and autonomy, being sensitive to the impact of social (peer) influences, attunement to descriptive and injunctive norms, and the availability of appealing alternatives. For some youth, the desire to protect one’s health conflicted with adolescent-typical impulses to experiment, fueling resistance to using substances. For others, experience of social influences and/or impulses toward novelty and experimentation tipped decision-making toward risk behaviors. Where influences moved youth toward risk, participants reported employing a range of behavioral and cognitive strategies to reconcile the dissonance they felt between the threat to health from substance use and their concern to be healthy, and avoid complications and flares (Table 3). Subthemes are outlined below.

**Table 3:** Theme 2. The adolescent stage.

Subtheme	Concept	Exemplar quote
Developmental impulses	Appetite for novelty and experimentation	I did [have fun drinking with my friends]. It was more fun because it was like the introduction to it, not because of the people I was doing it with. I was trying something new, and it was sort of adventurous. (18-year-old female with JIA) I figured I'll give [marijuana] a try, whatever. It was never like a pressure situation. It was just like I wanna see what it does to you. (17-year-old male with T1D)
	Reactance against perceived authority (psychological reactance)	…I was always told that I can’t [drink], so that made me want to do it more. (18-year-old female with JIA) For a lot of kids, it's like, the temptation to drink is because they're not allowed to, like their parents say “no.” (18-year-old female with T1D)
	Value for autonomous decision-making	It's possible that someone with [Rheumatoid Arthritis] isn't going to care as much about their body as I do and that's perfectly fine like, “you do you.” (18 year-old female with JIA) We know there are a lot of people that do[drugs] and I mean if they want to do it…[that is] their own business… I have nothing to do it with it…If you're gonna do that, then do it. But I'm not gonna do it. (19-year-old male with JIA) …there wasn't like any peer pressure or anything either. [Drinking] was like my decision I made on my own. (19-year-old male with JIA) Everyone has their own choices and opinions [about marijuana]. (19-year-old female with asthma)
Social and contextual influences	Perceived friend group norms	I'd say starting at the beginning of junior year [of high school] basically like everyone drinks like, if you don't drink you really can't socialize. (18-year-old female with JIA) …this must've been on my mind that other people were doing it (drinking), so I might as well try it. (19-year-old male with JIA)
	Observational learning	…after my friend had passed away–she had diabetes and she did have complications that were directly from drinking, so like that was partially why I like chose to not drink. (19-year-old female with T1D) …when [my cousin] got a little bit older she started to neglect [her diabetes] and not take her insulin, and she would drink a lot of alcohol and got into drugs and things like that. And now she has lost a lot of her vision and has gastroparesis and stuff like that that I don't want to have. (17-year-old female with T1D) …Every weekend [people] would [drink] and that would just be so normal to them, and I feel like they learn a lot doing it. (18-year-old female with JIA)
	Availability of attractive alternatives	Well, I have plenty of fun without alcohol itself and I just like to enjoy the situation. Like if we are having a fire, I will make a nice s'more or at least roast some marshmallows for someone else. (17-year-old male with T1D) It was one of those things where it was just like I was bored over summer and I wanted to do it (drink). (18-year-old female with JIA)
Balancing adolescent impulses and chronic disease concerns	Adjusting treatment regimen	I try really hard to take my methotrexate on the day that's like, the furthest from the day that I would be drinking. So like if I was drinking on a Saturday, I would try to take it on a Tuesday or Wednesday night. (18-year-old female with JIA) I have to give myself 60 units [of insulin] per beer…so say I'm heading out, I'll give myself 120 units, have a couple beers, wait a little bit, have some more units, have some more beers. It's kind of like that. (17-year-old male with T1D)
	Minimization of risk	…with all the technology now you really don't have to worry about [diabetes] that much [while drinking]. If you just have it in the back of your mind, then you're fine. I really just can live life normally. (18-year-old male with T1D) I’m just not like stupid about [drinking on methotrexate]…I wouldn’t like chug an insane amount. (18-year-old female with JIA) I keep [my drinking] under control pretty good. I'm not an alcoholic or anything so it doesn't affect [my diabetes] much. (17 year-old male with T1D)
	Substitution	…I have like tried pot instead [of drinking] and like even [my parents] were obviously not happy about that decision, but like understood that it wasn't like affecting my diabetes as much as like other decisions might have. (19-year-old female with T1D) I get high instead of getting drunk…Well, the diabetes–that's pretty much it…That’s pretty much the only reason [why I don’t drink]. (19-year-old male with T1D)

#### Developmental impulses

The appeal of novel experiences and an impulse for experimentation were drivers of substance use, especially initiation. Reactance against rules was, for some, a reinforcing factor. Across the sample, youth placed high value on autonomy which was central to personal decisions to abstain from as well as use substances. Youth evaluated their own and peer behaviors similarly; several described peer behaviors as reflecting “personal choice.” Preserving autonomy and self-determination and assuming a non-judgmental stance even for unhealthy behaviors are core values for this age group.

#### Social and contextual influences

Youth reported sensitivity to peer behaviors and norms. Associating with friends who use substances provided an opportunity to observe harms resulting from use and also to consider benefits; both positive and negative consequences of using were evident in stories youth shared about friends’ substance use. Participants also reported obtaining substances from friends, a factor contributing to use. A perceived lack of attractive social alternatives increased desire to use substances to relieve boredom or conform socially. Engagement and commitment to prosocial activities (e.g., academics) as well as availability of appealing sober social opportunities (e.g., watching movies with friends) supported decisions not to use.

#### Balancing adolescent impulses and chronic disease concerns

Participants employed a range of cognitive and behavioral strategies to reconcile the dissonance they experienced between the developmentally driven appeal of substance use–i.e., value placed on novelty, experimentation, sociability, and conformity–and the potential that substance use would jeopardize their health and/or contribute to disease exacerbations or flares. An area of acute dissonance reflected concern for potential risks stemming from simultaneous exposure to alcohol and alcohol-interactive medications. Youth took several behavioral steps to counter this risk. Some youth adjusted treatment regimens without clinical guidance, skipped medications, or spaced out the timing between taking a medication and using alcohol to avoid potentially harmful interactions. Those with T1D mentioned increasing the frequency of blood glucose monitoring and strategically adjusting their insulin and food intake to compensate for alcohol use. Substitution served as an additional behavioral strategy. Several participants opted for avoiding alcohol and using marijuana on select occasions; these youth perceived marijuana to be less health-compromising and non-interactive with medications. Cognitive strategies were evident too. To cope with concerns about their health, participants minimized perceived risks associated with substance use. Some rationalized that their level of alcohol use does not rise to the level of clinically significant abuse or a disorder–a cognitive strategy that, even if true, ignores any special risk from substance use for this group given their medical condition.

### Theme 3: Disease-specific substance use knowledge

Participants were curious about how substance use affects their condition and its treatment, indicating gaps in understanding. They were eager to discuss these issues during their specialty care visits and expressed clear preferences for content of messages and their delivery (Table 4). Subthemes are outlined below.

**Table 4:** Theme 3. Disease-specific substance use knowledge.

Subtheme	Concept	Exemplar quote
Information deficits	Questions and gaps in knowledge	I've always been told that [drinking] messes everything up but I don't know if [my blood sugar] goes high or low. (17-year-old male with T1D) …ultimately, how [does drinking] really affect Crohn’s? Because even though you see it online that this is what happens, you don’t know the actual bodily process of *why* that happens. (19-year-old female with IBD)
	Obtaining information on one’s own	I read–before–my bottles of medicine. It's like, "Do not take with alcohol." And I just went online, [to check] if it's good to drink alcohol with medicine, and basically it just said like, "No, don't [drink] because [the medicine] won't work." (16-year-old female with polymyositis) I Googled if there were any super adverse side effects [of drinking]…just to make sure there were no, "you're gonna die if you do this" or anything like that. (19-year-old female with IBD) [My pediatrician] said something about [how] my medication is absorbed through my liver…[and how drinking] can mess up the absorption of my medication…it made me a bit worried but when I asked my older friends, they said that if it’s once in a while…it’s probably not going to do anything. (18-year-old female with JIA).
Preferences for content and delivery of substance use messages	Factual and disease-specific	…[what] made an impression on me is probably the fact that the glucagon wouldn't work if I was severely intoxicated…I think that's the thing that I'd really say scared me the most because that's kind of a scary thought that like, you could die from drinking because your blood sugar was low and they had no way–and even in the hospital, they can give you IV's and stuff, but they really can't do much for you until the alcohol has been processed through your system. (18-year-old female with T1D) [The topic of drinking] should be integrated when [providers] talk to you about how exercise and stress and things like that may affect your blood sugar. They should include alcohol and say what it does…that way the patient will know if they do make the decision to drink. Then they know what happens to their blood sugar levels and they know it's affecting them rather than having them drink and then having something bad happen and they'll have to learn from their mistakes instead of knowing from the beginning. (17-year-old male with T1D)
	From the specialist	…there is no other person in my life or anyone's life that has Crohn’s or any disease that knows more about, not only the disease, but my experience with the disease than my [GI] doctor. He is the utmost figure of knowledge, so I always want to hear what my doctor has to say. (19-year-old female with IBD) Your diabetes doctor should know the most about the positive and negative side effects about [using substances with] diabetes…they should know the most and should have the most advice to give–the most solid advice to give–as opposed to like looking it up on the Internet … (19-year-old male with T1D) I would definitely look to [my gastroenterologist] for someone to give advice on how much [alcohol] I should be consuming because he knows best about how it would affect my body. (19-year-old female with IBD).
	Direct and honest	…laying out all of the information [about drugs and alcohol] is probably the best thing to do…not trying to hold back any information because it might be scary. I think it's just best to say everything, even if it's unlikely to occur…it's best to be the most informed about any situation that could occur. (19-year-old female with IBD-associated arthritis) I mean sometimes because we're underage, pediatricians and children's doctors just kind of sugarcoat it kind of thing, and I'd rather someone just tells me exactly what's happening. (19-year-old female with JIA)
	Perceived permission	[My endocrinologist] says, ‘Stay away from rum. Scotch and whiskey are okay.’ …I definitely try to follow that rule. (18-year-old male with T1D). I mean [my doctor] realizes every 17-year-old is going to drink–it's just kind of bound to happen. (17-year-old male with T1D)
	Humanize the message	I feel like stories or like examples from your life or someone you know is kind of a good thing to tell people because then you know it’s real, like it actually happens. (16-year-old female with JIA) …because my doctor has diabetes, she's not just telling stuff she read in a book somewhere; she's telling me her personal stories…It means more when it's personal. (19-year-old male with T1D)
	Sensitive to developmental context and social pressures	I know doctors usually aren't our age but I feel like it'd be much more helpful to try to get on the level that like teens are at…most doctors now don't really know what kind of peer pressure is out there and stuff like that so I feel like it'd be more helpful to be like, "Well, I know the situation you're in and I know that you're being pressured to do these things and whatnot but you really have to not do that." (19-year-old female with IBD-associated arthritis)
	Private and confidential	…the problem is, though, that especially in [specialty care for] pediatrics, most of the time the kid’s parent is in [the clinic room] …I wish that a doctor could ask a parent to step out for a minute or something and be like, "Is there anything else you want to talk to me about?" That way a kid can feel safe. (19-year-old female with IBD) I mean no offense to [my rheumatologist], but I felt like he should have kind of like knew not to ask [about marijuana use] in front of a parent if he wanted a truthful answer. (16-year-old female with JIA)

#### Information deficits

Many participants indicated that substance use harms are not clearly or consistently discussed as part of subspecialty medical care. Consequently, participants had unanswered questions about the impact of substance use on their condition and its treatment.

To lessen these gaps in understanding, many youth independently sought information, including through online sources. Some were unconvinced by what they considered to be a cursory discussion of harms held with their physician and sought confirmatory or supplemental information from alternative sources. Inattention to these issues within healthcare settings created room for inaccurate or misguided information from peers or others to shape participant beliefs and behaviors.

#### Preferences for content and delivery of substance use messages

Youth voiced strong preferences for bridging the topics of substance use and their chronic condition in a manner that is factual and specific. Participants asserted that this information would be best delivered by their specialist who they considered the expert on their body and condition.

A common theme was a preference for a direct and honest discussion about the disease-specific risks of substance use without “any gray area” or “sugar-coating.” When clinicians did not directly articulate the risks or recommend cessation, participants sometimes interpreted this as tacit granting of permission to use substances. Youth appreciated when providers demonstrated sensitivity to their developmental context and the social pressures they face. Further, communicating real-life examples of substance use harms served to humanize messages about substance use. Finally, many youth expressed that time alone with their provider and assurances of confidentiality were necessary to creating a safe space for honest discussion and disclosure.

## Discussion

In this investigation, we found that among youth with a chronic illness, decision-making about substance use is integrally related to their experiences living with and managing their chronic condition. Awareness of having a chronic illness provides an omnipresent frame of reference which bears on the many calculations youth make about how to manage their condition and assess safety and risk. This frame and the life experiences of this group may prepare them to perceive substance use-related health risks as serious or severe–a vital toehold for prevention. The developmental stage of adolescence and the appeal of novelty and experimentation play a role in risk perception and behavioral decision-making, as may the perceived benefits of using substances (particularly marijuana) to address symptoms and treatment side effects. However findings suggest that these factors may not outweigh the influence of messages that speak directly to how substance use can amplify medical vulnerabilities. Rather, clear guidance and messaging about these issues are welcomed and valued by youth. While prevention efforts will need to consider the push-pull among these factors, findings suggest that youth are primed to prioritize behaviors aligned with maintaining disease control and symptom quiescence over those that reinforce novelty and sociability. Indeed, the deeply personal lived experience of a chronic illness and attendant disease and treatment burdens create the potential to harness affective, experiential and deliberative components of risk perception [59] for impactful interventions. Findings overall bear on development of clinical services to prevent and reduce substance use among medically vulnerable youth, and provide insight into how dimensions of behavioral theories might be adapted to guide such efforts by emphasizing messaging that speaks to the unique concerns and experiences of these youth.

Several issues may be particularly salient when intervening with this group. These include delivery of factual and disease-specific messages about substance use risks and guidance around the importance of avoiding substance use. Youth favored this type of guidance and its absence may leave cognitive “wiggle room” sufficient to enabling *un*healthy choices. Overall, narratives revealed tendencies toward denial, minimization of risk, and an inflated sense of control over risk management. Lack of specific information also provides a context in which youth may perceive near term benefits of use to relieve symptoms, in the case of marijuana, without access to specific information concerning risks to health long-term.

YCMC perceived a disconnect between substance use behaviors and their value for health–relief from cognitive dissonance was sought at times by efforts to alter treatment regimens to accommodate drinking (i.e., changing dosing, scheduling, and adherence to medications), as opposed to choosing abstinence. Helping youth understand and internalize substance use risks and prioritize concerns to protect their health over social and developmental influences may foster consistent healthy decision-making and better outcomes. Prior research has shown that poor knowledge about the potential for alcohol interactions with medications and laboratory tests is associated with sharply elevated risks of alcohol use and binge drinking among YCMC as well as regular medication non-adherence [26,31] Addressing these issues as part of a targeted preventive intervention may offer valuable guidance that contributes to health protecting choices and actions.

Learning how to effectively harness medically vulnerable adolescents’ cognitions, motivations, and health concerns to support positive decision making may be especially important. Findings from developmental neuroscience suggest the difficulty of countermanding the socially and physiologically reinforcing effects of substance use behaviors on reward pathways instantiated in the adolescent brain [60–63] which mature prior to those that center on consequential thinking [10,60]. In this context, it is vitally important to develop preventive messages that are patient-centered and persuasively impact top concerns and motivations of medically vulnerable youth; identifying key touch points may offer the traction necessary for messages to align with established values and priorities to prevent disease exacerbations by avoiding substance use and motivating healthy choices.

YCMC prefer information that connects substance use risks to impacts on their condition and favor messaging delivered by health experts with whom they have a trusting relationship. Locating psycho-educational prevention in pediatric specialty care was highly acceptable and preferred by these youth who also reported they value being asked direct questions about health risk behaviors in confidence, consistent with reports of healthy teens [64–66] and teens with chronic medical conditions [67]. Thus, provision of confidential verbal screening with unambiguous questions followed by clear advice about avoiding or resisting substance use while acknowledging the potential for conflicting social developmental pressures may be key features of a successful clinical conversation that aligns with national priorities for adolescent-friendly clinical preventive services [68]. Participants reported that discussions about substance use are not occurring during subspecialty visits or feel perfunctory and unsatisfying. This finding mirrors the low rates of alcohol screening and counseling among pediatric subspecialists found in the most recently published survey reports [69,70].

Qualitative interviews undertaken for this study afford insight into motivations and decision-making around substance use and elucidate aspects of substance use screening and messaging that are valued by medically vulnerable youth. Findings may inform preventive interventions and build on epidemiologic reports about YCMC risks [26,27,71]. In this context several limitations of this research merit mention. The sample represents a mix of conditions and patient background characteristics, however interviews were conducted with patients at a single institution and findings may not generalize. The sample is predominantly white and most participants reported parents with some college education which, while consistent with clinic demographics, could exclude youth from minority and other vulnerable backgrounds. Youth understanding and interpretation of past events are especially relevant to constructing salient health messages and capturing youth perspectives was the focus of this investigation. Nevertheless, recall and social desirability biases can affect the accuracy of participant reports, especially in qualitative work. For these reasons interviewers prefaced each interview with the open-ended aim of the study, offered assurances of confidentiality, and took a curious, nonjudgmental stance.

## Conclusions

Findings point to the need for clinicians—especially pediatric specialists—to provide substance use education and guidance to YCMC that connects risks and recommendations to patient-centered disease-specific concerns and goals. Nuanced interventions are needed that address the complexity of navigating chronic disease management and adolescent-typical impulses and pressures. Development and evaluation of tailored, targeted prevention messages and programs that engage youth in thoughtful, respectful, and impactful discussions about their risks and choices are important next steps to protecting the health of medically vulnerable youth.

## Supporting information

S1 File Interview guide. This is the semi-structured interview guide.
(DOCX)

S2 File Excerpts of quotes relevant to study. This is the listing of quotes used in the study.
(PDF)

## References

[1] UNFPA. The State of World Population 2014: “Adolescents, Youth and the Transformation of the Future.” New York, NY: 2014.

[2] United States Census Bureau. QuickFacts, United States 2016. https://www.census.gov/quickfacts/fact/table/US/PST045216.

[3] Van Cleave J, Gortmaker SL, Perrin JM. Dynamics of obesity and chronic health conditions among children and youth. JAMA J Am Med Assoc 2010;303:623–30. 10.1001/jama.2010.104 20159870

[4] U.S. Department of Health and Human Services, Health Resources and Services Administration, Maternal and Child Health Bureau. The National Survey of Children with Special Health Care Needs Chartbook 2009–2010 2013.

[5] Wisk LE, Weitzman ER. Expectancy and Achievement Gaps in Educational Attainment and Subsequent Adverse Health Effects Among Adolescents With and Without Chronic Medical Conditions. J Adolesc Heal 2017;61:461–70. 10.1016/j.jadohealth.2017.04.006 28734632 PMC5610930

[6] Wisk LE, Weitzman ER. Social Relationships and Associated Health Effects among Adolescents with and without Chronic Medical Conditions. Soc. Adolesc. Heal. Med. Heal. Annu. Meet., New Orleans, LA: 2017. 10.1016/j.jadohealth.2017.04.006 PMC5610930 28734632

[7] Compas BE, Jaser SS, Dunn MJ, Rodriguez EM. Coping with chronic illness in childhood and adolescence. Annu Rev Clin Psychol 2012;8:455–80. 10.1146/annurev-clinpsy-032511-143108 22224836 PMC3319320

[8] Kann L, Kinchen S, Shanklin SL, Flint KH, Kawkins J, Harris WA, et al Youth risk behavior surveillance—United States, 2013. MMWR Surveill Summ 2014;63:1–168. doi: ss6304a1 [pii]. 24918634

[9] Hingson RW, Heeren T, Winter MR. Age at Drinking Onset and Alcohol Dependence: Age at Onset, Duration, and Severity. Arch Pediatr Adolesc Med 2006;160:739–46. 10.1001/archpedi.160.7.739 16818840

[10] Galvan A, Hare TA, Parra CE, Penn J, Voss H, Glover G, et al Earlier development of the accumbens relative to orbitofrontal cortex might underlie risk-taking behavior in adolescents. J Neurosci 2006;26:6885–92. 10.1523/JNEUROSCI.1062-06.2006 16793895 PMC6673830

[11] Osilla KC, Pedersen ER, Ewing BA, Miles JN, Ramchand R, D’Amico EJ. The effects of purchasing alcohol and marijuana among adolescents at-risk for future substance use. Subst Abus Treat Prev Policy 2014;9:1–10. 10.1186/1747-597x-9-38 25231097 PMC4177688

[12] Marschall-Lévesque S, Castellanos-Ryan N, Vitaro F, Séguin JR. Moderators of the Association Between Peer and Target Adolescent Substance Use. Addict Behav 2014;39:48–70. 10.1016/j.addbeh.2013.09.025 24183303 PMC3858300

[13] Griffin KW, Botvin GJ. Evidence-Based Interventions for Preventing Substance Use Disorders in Adolescents. Child Adolesc Psychiatr Clin N Am 2010;19:505–26. 10.1016/j.chc.2010.03.005 20682218 PMC2916744

[14] Substance Abuse and Mental Health Services Administration, Center for Behavioral Health Statistics and Quality. The NSDUH Report: Trends in Adolescent Substance Use and Perception of Risk from Substance Use 2013. 27656743

[15] Goldberg JH, Halpern-Felsher BL, Millstein SG. Beyond invulnerability: the importance of benefits in adolescents’ decision to drink alcohol. Health Psychol 2002;21:477–84. 10.1037/0278-6133.21.5.477 12211515

[16] Small SA, Silverberg SB, Kerns D. Adolescents’ perceptions of the costs and benefits of engaging in health-compromising behaviors. J Youth Adolesc 1993;22:73–87. 10.1007/BF01537905

[17] Petraitis J, Flay BR, Miller TQ. Reviewing theories of adolescent substance use: organizing pieces in the puzzle. Psychol Bull 1995;117:67–86. 10.1037/0033-2909.117.1.67 7870864

[18] World Health Organization. Global Status Report on Noncommunicable Diseases 2010. Geneva, Switzerland: World Health Organization; 2011.

[19] Nikolic IA, Stanciole AE, Zaydman M. Chronic Emergency: Why NCDs Matter. Heal Nutr Popul Discuss Pap 2011.

[20] Engelgau MM, El-Saharty S, Kudesia P, Rajan V, Rosenhouse S, Okamoto K. Capitalizing on the Demographic Transition: Tackling Noncommunicable Diseases in South Asia. Washington, D.C.: The World Bank; 2011.

[21] Bloom DE, Cafiero ET, Jané-Llopis E, Abrahams-Gessel S, Bloom LR, Fathima S, et al. The Global Economic Burden of Noncommunicable Diseases. Geneva, Switzerland: 2011.

[22] World Bank. The Growing Danger of Non-Communicable Diseases: Acting now to reverse course. Washington, D.C.: 2011.

[23] Patton GC, Coffey C, Cappa C, Currie D, Riley L, Gore F, et al Health of the world’s adolescents: A synthesis of internationally comparable data. Lancet 2012;379:1665–75. 10.1016/S0140-6736(12)60203-7 22538181

[24] World Health Organization. Tobacco Free Initiative. Geneva, Switzerland: 2012.

[25] Popkin BM, Adair LS, Ng SW. Global Nutrition Transition and the Pandemic of Obesity in Developing Countries. Nutr Rev 2012;70:3–21. 10.1111/j.1753-4887.2011.00456.x 22221213 PMC3257829

[26] Weitzman ER, Ziemnik RE, Huang Q, Levy S. Alcohol and Marijuana Use and Treatment Nonadherence Among Medically Vulnerable Youth. Pediatrics 2015;136:450–7. 10.1542/peds.2015-0722 26668849 PMC4552090

[27] Wisk LE, Weitzman ER. Substance use patterns through early adulthood: Results for youth with and without chronic conditions. Am J Prev Med 2016;51:33–45. 10.1016/j.amepre.2016.01.029 27039116 PMC4914415

[28] Substance Abuse and Mental Health Services Administration. The Dawn Report: Alcohol and Drug Combinations Are More Likely to Have a Serious Outcome Than Alcohol Alone in Emergency Department Visits Involving Underage Drinking 2014.

[29] Nash AA, Britto MT, Lovell DJ, Passo MH, Rosenthal SL. Substance use among adolescents with juvenile rheumatoid arthritis. Arthritis Care Res 1998;11:391–6. 10.1002/art.1790110510 9830883

[30] Barnard K, Sinclair JMA, Lawton J, Young AJ, Holt RIG. Alcohol-associated risks for young adults with Type1 diabetes: a narrative review. Diabet Med 2012;29:434–40. 10.1111/j.1464-5491.2012.03579.x 22248115

[31] Weitzman ER, Magane KM, Wisk LE, Allario J, Harstad E, Levy S. Alcohol Use and Alcohol-Interactive Medications Among Medically Vulnerable Youth. Pediatrics 2018;142:e20174026 10.1542/peds.2017-4026 30228168 PMC6317570

[32] Wong NCH, Cappella JN. Antismoking threat and efficacy appeals: Effects on smoking cessation intentions for smokers with low and high readiness to quit. J Appl Commun Res 2009;37:1–20. 10.1080/00909880802593928 20046966 PMC2680609

[33] Substance Abuse and Mental Health Services Administration. Prevention Approaches 2016. https://www.samhsa.gov/capt/practicing-effective-prevention/prevention-approaches (accessed October 30, 2017).

[34] Murthy VH, Rudd R, Stahre M, Botticelli M, Grant B. Facing Addiction in America: The Surgeon General’s Report on Alcohol, Drugs, and Health. 2016 10.1001/jama.2016.18215 28252892

[35] Substance Abuse and Mental Health Services Administration. Focus on Prevention. HHS Publication No. (SMA) 10–4120 2017.

[36] Glanz K, Bishop DB. The Role of Behavioral Science Theory in Development and Implementation of Public Health Interventions. Annu Rev Public Health 2010;31:399–418. 10.1146/annurev.publhealth.012809.103604 20070207

[37] Griffin KW, Botvin GJ. Evidence-Based Interventions for Preventing Substance Use Disorders in Adolescents. Child Adolesc Psychiatr Clin N Am 2010;19:505–26. 10.1016/j.chc.2010.03.005 20682218 PMC2916744

[38] Arain M, Haque M, Johal L, Mathur P, Nel W, Rais A, et al Maturation of the adolescent brain. Neuropsychiatr Dis Treat 2013;9:449–61. 10.2147/NDT.S39776 23579318 PMC3621648

[39] Dahl RE. Adolescent brain development: a period of vulnerabilities and opportunities. Keynote address. Ann N Y Acad Sci 2004;1021:1–22. 10.1196/annals.1308.001 1021/1/1 [pii]. 15251869

[40] Simons-Morton BG, Farhat T. Recent findings on peer group influences on adolescent substance use. J Prim Prev 2010;31:191–208. 10.1007/s10935-010-0220-x 20614184 PMC3313483

[41] Kobus K. Peers and adolescent smoking. Addiction 2003;98 Suppl 1:37–55. 12752361 10.1046/j.1360-0443.98.s1.4.x

[42] Holder HD. Supply Side Approaches to Reducing Underage Drinking: An Assessment of the Scientific Evidence In: National Research Council (US) and Institute of Medicine (US) Committee on Developing a Strategy to Reduce and Prevent Underage Drinking, Bonnie R, O’Connell M, editors. Reducing Underage Drink. A Collect. Responsib., Washington DC: National Academies Press (US); 2004. 20669473

[43] Christiansen BA, Goldman MS, Inn A. Development of alcohol-related expectancies in adolescents: separating pharmacological from social-learning influences. J Consult Clin Psychol 1982;50:336–44. 10.1037/0022-006X.50.3.336 7096736

[44] Christiansen BA, Smith GT, Roehling P V., Goldman MS. Using alcohol expectancies to predict adolescent drinking behavior after one year. J Consult Clin Psychol 1989;57:93–9. 10.1007/s11121-008-0090-0 2925979

[45] Chen MJ, Grube JW, Madden PA. Alcohol expectancies and adolescent drinking: differential prediction of frequency, quantity, and intoxication. Addict Behav 1994;19:521–9. 10.1016/0306-4603(94)90007-8 7832010

[46] Olds RS, Thombs DL. The relationship of adolescent perceptions of peer norms and parent involvement to cigarette and alcohol use. J Sch Health 2001;71:223–8. 10.1111/j.1746-1561.2001.tb01322.x 11512489

[47] Beck KH, Treiman KA. The relationship of social context of drinking, perceived social norms, and parental influence to various drinking patterns of adolescents. Addict Behav 1996;21:633–44. 10.1016/0306-4603(95)00087-9 8876762

[48] Aas H, Klepp K. Adolescents’ alcohol use related to perceived norms. Scand J Psychol 1992;33:315–25. 10.1111/j.1467-9450.1992.tb00920.x 1287824

[49] Stuckey H. The second step in data analysis: Coding qualitative research data. J Soc Heal Diabetes 2015;03:007–010. 10.4103/2321-0656.140875

[50] Gibbs GR. Analyzing Qualitative Data. SAGE Publications; 2007 10.4135/9781849208574.

[51] Miles MB, Huberman MA. Qualitative Data Analysis. SAGE Publications; 1994.

[52] Taylor SJ, Bogdan R. Introduction to Qualitative Research Methods. 3rd ed Hoboken, NJ: John Wiley & Sons, Inc; 1998.

[53] Schwenk HT, Lightdale JR, Arnold JH, Goldmann DA, Weitzman ER. Coping with college and inflammatory bowel disease: implications for clinical guidance and support. Inflamm Bowel Dis 2014;20:1618–27. 10.1097/MIB.0000000000000124 25105948

[54] Berry JG, Kusminsky M, Foley SM, Hobbs N, Queally JT, Bauer SB, et al Strategic directions for transition to adulthood for patients with spina bifida. J Pediatr Neurol 2013;11:211–20. 10.3233/JPN-130624

[55] Corbin JM, Strauss A. Grounded theory research: Procedures, canons, and evaluative criteria. Qual Sociol 1990;13:3–21. 10.1007/BF00988593

[56] Strauss A, Corbin J. Handbook of Qualitative Research. Thousand Oaks, Sage Publications; 1994.

[57] Bandura A. Social foundations of thought and action: A social cognitive theory. Englewood Cliffs, NJ: Prentice-Hall, Inc; 1986.

[58] Tong A, Sainsbury P, Craig J. Consolidated criteria for reporting qualitative research (COREQ): a 32-item checklist for interviews and focus groups. Int J Qual Heal Care 2007;19:349–57. 10.1093/intqhc/mzm042 17872937

[59] Ferrer RA, Klein WMP, Persoskie A, Avishai-Yitshak A, Sheeran P. The Tripartite Model of Risk Perception (TRIRISK): Distinguishing Deliberative, Affective, and Experiential Components of Perceived Risk. Ann Behav Med 2016;50:653–63. 10.1007/s12160-016-9790-z 26961206

[60] Sharma A, Morrow JD. Neurobiology of Adolescent Substance Use Disorders. Child Adolesc Psychiatr Clin N Am 2016;25:367–75. 10.1016/j.chc.2016.02.001 27338961

[61] Chein JM, Albert D, O’Brien L, Uckert K, Steinberg L. Peers increase adolescent risk taking by enhancing activity in the brain’s reward circuitry. Dev Sci 2011;14:F1–10. 10.1111/j.1467-7687.2010.01035.x 21499511 PMC3075496

[62] Steinberg L. A Social Neuroscience Perspective on Adolescent Risk-Taking. Dev Rev 2008;28:78–106. 10.1016/j.dr.2007.08.002 18509515 PMC2396566

[63] U.S. Department of Health and Human Services (HHS), Office of the Surgeon General. The Surgeon General’s Report on Alcohol, Drugs, and Health. Washington, DC: 2016. 28252892

[64] Britto MT, Slap GB, DeVellis RF, Hornung RW, Atherton HD, Knopf JM, et al Specialists Understanding of the Health Care Preferences of Chronically Ill Adolescents. J Adolesc Heal 2007;40:334–41. 10.1016/j.jadohealth.2006.10.020 17367726

[65] Cheng TL, Savageau JA, Sattler AL, DeWitt TG. Confidentiality in health care. A survey of knowledge, perceptions, and attitudes among high school students. JAMA 1993;269:1404–7. 8441216 10.1001/jama.269.11.1404

[66] Ginsburg KR, Slap GB, Cnaan A, Forke CM, Balsley CM, Rouselle DM. Adolescents’ Perceptions of Factors Affecting Their Decisions to Seek Health Care. Jama 1995;273:1913–8. 10.1001/jama.1995.03520480033036 7783300

[67] Britto MT, DeVellis RF, Hornung RW, DeFriese GH, Atherton HD, Slap GB. Health care preferences and priorities of adolescents with chronic illnesses. Pediatrics 2004;114:1272–80. 10.1542/peds.2003-1134-L 15520107

[68] Harris SK, Aalsma MC, Weitzman ER, Garcia-Huidobro D, Wong C, Hadland SE, et al Research on Clinical Preventive Services for Adolescents and Young Adults: Where Are We and Where Do We Need to Go? J Adolesc Heal 2017;60:249–60. 10.1016/j.jadohealth.2016.10.005 28011064 PMC5549464

[69] Britto M, Garrett J, Dugliss M, Johnson C, Majure J, Leigh M. Preventive services received by adolescents with cystic fibrosis and sickle cell disease. Arch Pediatr Adolesc Med 1999;153:27–32. 9894996 10.1001/archpedi.153.1.27

[70] Britto M, Rosenthal S, Taylor J, Passo M. Improving rheumatologists’ screening for alcohol use and sexual activity. Arch Pediatr Adolesc Med 2000;154:478–83. 10.1001/archpedi.154.5.478 10807299

[71] Levy S, Dedeoglu F, Gaffin JM, Garvey KC, Harstad E, MacGinnitie A, et al A Screening Tool for Assessing Alcohol Use Risk among Medically Vulnerable Youth. PLoS One 2016;11:e0156240 10.1371/journal.pone.0156240 27227975 PMC4882018

