# Supplementary material for: Perspectives on substance use among youth with chronic medical conditions and implications for clinical guidance and prevention: A qualitative study
Source: PLoS One. 2019 Jan 23;14(1):e0209963. doi: 10.1371/journal.pone.0209963 (PMC6343873; doi:10.1371/journal.pone.0209963)
Supplement: S1 File — This is the semi-structured interview guide. (DOCX) [file pone.0209963.s001.docx]

**S1. Interview Guide**

**Background Information, Healthcare Management, Disease Identity**

1. So tell me a little more about you. What grade are you in? What do you like to do in your spare time? *Probes*: Do you do any extracurricular activities? Play any sports? What do you do for fun?
   1. For many kids, their [condition] affects their day-to-day life. Does your [asthma/CF/arthritis/diabetes/IBD] ever get in the way of doing these things? Are there ways that your condition affects aspects of your everyday life?
2. Have you ever had to choose your health over some other activity? (If applicable) Can you give me an example of a recent time where you had to do that?
3. What is your medication regimen?
4. How much is [condition] a part of your life? What about in terms of your identity – how you define yourself? *Probe for the way in which it’s a part of their identity/self-definition – how it has shaped them.*
5. What does being healthy mean to you? What stands in the way of you being healthy?
6. Do you ever feel burned out by having to manage your [condition]? *Probe*: Tell me about that.
7. Do you feel like you’ve come to terms with having [condition]? Was there a time you realized you were in this for life, that you had to accept having [condition]? *Probe*: What sparked it? What shifted? How would you motivate someone else?

**Social Context**

1. Does your [chronic condition] ever make you feel different from your friends (e.g., in terms of your outlook on things or how you approach life)? In what ways? *Explore illness identity. Probe for perception that they are older/more mature/understand the world, sense of isolation from peers and potential for alcohol/partying to connect them to more age typical behaviors.*
2. Thinking about how your [condition] has affected your life, do you think your experience of “the teen years” is different from that of your friends in terms of the pace of growing up (faster, slower, in sync)? *Probe for tempo of adolescence.*

**Substance Use: Knowledge, experience, social factors**

1. Alcohol
   1. Is drinking “a thing” for your close friends? How about you? *Probe for the role of alcohol in friendship*:
       (If yes) Did you consciously decide to become friends with people who drink or did the drinking start while you were already friends? Has their decision to drink affected your friendship at all? How?
       (If no) Did you consciously choose to avoid being friends with people who drink or has it just not come up? Tell me about that.
   2. What is your experience with drinking? (If in college) What was it like transitioning to college with your [condition] and navigating drinking situations? *Probe* (if they drink): How often do you drink? What grade were you in when you first started drinking? Is that when your friends started? Tell me the story of when you first started drinking. Do you recall trying to wait? *Explore what shifted.*
      1. (If participant drinks)
         1. In what situations do you drink (with friends, at parties)? *Probe*: Do you drink/party with friends who know about or have your condition?
         2. Have you ever had a problem because of your drinking, a general problem (e.g., problem with the law, with parents, at school; blacking out, an accident) or one around your [chronic condition]? Have you ever gone to the emergency room because of drinking? *Probe:* (If yes) Tell me about that. Describe the situation. What was the most concerning aspect of this for you/what were you most worried about? Did you talk to your doctor about this? Did this experience change the way you think about alcohol? In light of this experience, would you do anything differently in the future?
         3. What precautions do you take when drinking, if any? How do you manage your medication(s)/sugar levels in drinking situations? *Probes*: Is the medication still in your body while you’re drinking? Do you change the timing of your medication or skip taking it when you know you’re going to drink? Have you ever forgotten to take it the next morning?
         4. What is it like drinking with your [condition]? *Probe*: Consider how you versus your friends approach drinking situations. Is there anything special you have to consider because of your [condition]? Does drinking affect your ability to tune into your body/detect body cues important to managing your condition? How? (If applicable) Where did you get that information?
         5. What happens to you when you drink on your medication(s)? *Probe:* What information do you have, if any, about what might happen if you drink on your medication(s)? *Probe for what might happen around effectiveness of medications and side effects and if that is something they are concerned about.* Where did you get that information? Do you have any concerns around drinking on your medication(s)? (If yes) What concerns do you have?
         6. What are your motivations to drink? *Explore motivations*. Does your friends [drinking or not drinking] affect your own experience with drinking/decision to drink? Tell me about that.
      2. (If participant doesn’t drink)
         1. What motivates you to not drink? What are some things that stop you from drinking? Does your friends’ [drinking or not drinking] affect your decision to not drink? Tell me about that.
         2. Do your friends know why you don’t drink? *Probe*: Are they understanding? Do your friends know you don’t drink because of [above reason]?
   3. Do you feel like you have an alternative to drinking? *Probes*: What do you do instead? Are there things you feel like you can do in your free time, such as social activities or hobbies, aside from drinking?
   4. Have you ever felt peer pressure to drink? *Probe*: Tell me about it.
   5. Have you ever been in a situation where you had to say no to drinking? *Probes*: Would you tell me about that? What influenced your decision to drink versus not drink? *Explore motivations.* Looking forward, what might you do differently?
2. Marijuana
   1. Do any of your friends ever smoke marijuana?
   2. How about you? Do you ever smoke marijuana?
   3. (Repeat alcohol questions for marijuana as applicable)
   4. What are your thoughts on using marijuana as medicine?
3. (If participant uses more than one substance) Which do you prefer? *Probe for drug substitution – if they ever pick one over the other.* Do you tolerate one more because of your [chronic condition]?

**Messaging** **in the Clinical Setting, Interplay Between Substance Use & Condition, and Information Needs**

1. What type of people do you see in the [specialty] clinic (i.e., physician, nurse practitioner, psychologist)?
2. Think about the [provider(s)] that you see in the [specialty] clinic. Have you ever had any conversations with them about drinking?
   1. (If yes) Describe the conversation(s). Was this the first time you were asked? How did it play out? Was your parent in the room? *Probes*: What did you think of the message they were trying to send (get at how the message was received—was it ignored or did it have an impact)? Did it make an impression on you? What did you like or dislike about their message? What did you think of the timing of their message – too early, too late, just right?
       *Comfort level*: How comfortable are you talking to your [specialty care provider] about these things? What plays into your comfort level (or lack thereof)?
       *Willingness to share*: How open/honest are you with your [specialty provider] about your drinking experiences? *Probe*: What affects your willingness to share?  *Feeling informed*: Do you feel informed about how drinking affects all the different aspects of your [condition]?
       *Integration*: Do you feel like the topic of drinking and other drugs is integrated into discussion about managing your [condition] or do you feel like it’s kept separate?
   2. (If no)
       *Comfort level*: How comfortable would you feel talking to your [specialty provider] about these things?
       *Willingness to share*: How open/honest are you willing to be with your [specialty provider]?
       *Feeling informed*: Do you feel informed about how drinking affects all the different aspects of your [condition] (tweak as applicable)?
       *Integration*: Do you feel like the topic of drinking and other drugs should be integrated into conversations about managing your [condition] or do you feel like it should be kept separate?
   3. (If participant uses other drugs, repeat b for other drugs)
3. In your opinion, how important is it to talk to your [specialty provider] about these issues compared to someone else? *Probes:* What makes this [specialty provider] an important person to talk to about these issues?
4. How do you want this conversation to happen in the clinic? What would make it effective? *Probe*: Think about messages you’ve heard from other people about alcohol or other drugs – were they effective/convincing/did the message stick and make an impact? (Yes) What made them effective? (No) What could make them more effective?
5. Have you ever looked up information on how alcohol or other drugs would affect your [condition] or its management/treatment?
6. If you were to ask your [specialty provider] anything about alcohol or other drugs, what questions would you ask, if anything? What are you curious about?

**Close**

1. Is there anything you want to share that I have not asked about?

Thank you for your time and your willingness to discuss these issues, your thoughts and stories are valuable!
