# Supplementary material for: Perspectives on substance use among youth with chronic medical conditions and implications for clinical guidance and prevention: A qualitative study
Source: PLoS One. 2019 Jan 23;14(1):e0209963. doi: 10.1371/journal.pone.0209963 (PMC6343873; doi:10.1371/journal.pone.0209963)
Supplement: S2 File — This is the listing of quotes used in the study. (PDF) [file pone.0209963.s002.pdf]

Excerpts of transcripts relevant to study

…I've only really lived life with [T1D]…so I don't really know what I'm missing. I mean, lows are scary. That's the main [concern]. Because I don't want to like- like when you're low you can just like pass out like if you're too low playing sports or something. (18-year-old male with T1D)

…[Diabetes is] a huge part of my life. I wake up every morning, I test my blood sugar. Like depending on that I'm either feeling awful or great if it's high or low or perfect…my whole life kind of revolves around trying to keep it as good as I can…I'm not trying to like keep it a secret but I definitely don't try to like put it out there that I'm diabetic. Like most of the people I've worked with for like over a year didn't even know I was diabetic. Not that I didn't want to tell them, it's just because it didn't come up and I keep it to myself. (19-year-old male with T1D)

I don't play sports at all… [On weekends or after school] I play computer games and I read. I mean I grew up wanting to do different stuff but just never being able to do it [because of my arthritis]. Like I wanted to do gymnastics when I was little. But you know like I think if I didn't have arthritis I'd be doing different things. But I grew up with arthritis so I don't really know. In general, it doesn't really affect me but it's because my life has sort of been shaped around it because I grew up with it. (17-year-old female with JIA)

Like if I get a job and it involves a lot of walking around, I want to try to avoid that, but that's the main thing. I just want to get a job where I know I'm going to be able to work every day and get my paychecks and be a dependable worker and not have to miss days because of my arthritis. When it was worse [my mom] was like, "I don't know how you're going to work." And I was like, "I'm going to try to control it" because I've missed a lot of days of school in the past. Like, I've missed 60 days because of arthritis. It was a little hectic. (16-year-old male with JIA)

When Crohn's first hit me, it was kind of during a time when I was becoming a teen, so I didn't really mature as much then. But once I wasn't affected by the Crohn's so much, then I kind of went through it faster… when I'm not feeling well or I'm stuck in the hospital, I'm not really going to get a chance to change my outlook on life or whatever. But then after all the experiences, when I got through it, that kind of made my personal view different. (17-year-old male with Crohn's)

A lot of the time I didn't feel like necessarily going out to do anything [because of my health]...if I just really wasn't feeling up to it, I'd tell [my friends] "I can't" or "I'm feeling really sick."… I think before we really knew what was happening there might have been a time or something where- I don't know if anyone said it straight out- I kind of felt like maybe people were doubting whether or not I was actually sick all the time. (17-year-old male with IBD)

Being healthy for [my friends] is just exercising and eating healthy. Whereas for me it's having the full energy to do stuff and make sure there are no flares. I mean it [gets in the way of my life] sometimes. It usually just makes me irritated. Usually I just try to have a really low key day

when that’s going on. So for the most part if I sense that it’s something that's happening, I'll just hang at home and watch movies or something so I'll be in the comfort of my own home all day. (19-year-old female with IBD)

To me I guess [being healthy] would be just being able to...kinda just do your everyday life without something getting in the way all the time. Because I've kind of come to the conclusion that I get more like infections and stuff easier than other kids so I really can't say that's like a healthy -- and I've kind of just come to the conclusion that if you can stay on your daily thing without something in the way, then I think I would consider that to be healthy. (19-year-old female with JIA)

I think healthy is kind of like when your body is working the way it should. Like you're not sick, you can do whatever you need to do in a day without an issue. (18-year-old male with T1D)

…It's definitely hard to cope with but I think that just making goals for yourself and understanding that you will have to go through, you know, terrible medications with awful side effects. It's just necessary because the disease itself is more debilitating than any of the medications. So you kind of just have to get through everything and take care of yourself in order to, I don't know, just function, really. [It’s hard to] be realistic all the time. Like that's really difficult to deal with but just something you have to do. You just have to realize that things can get better but you have to take all the medications that are prescribed to you...I think I just realized like how severe the illness was. (19-year-old female with IBD-associated arthritis)

...I had a decision to make: that I could let diabetes run my life or I could run my life and then just let diabetes tag along. So that was a route that I chose and my parents were really good about that like they never said no to certain things. It wasn't like, "No, you can't do that, you're diabetic." It was "You want to run around on the playground? Okay, we'll deal with it." So as far as I can remember diabetes never got in the way so it's not something that stops me from doing anything that I want to do. Anything I want to do, my diabetes comes along. (18-year-old female with T1D)

[I’m] probably honestly more...mature [than my peers]. I think because I had to grow up at such a young age. And I find myself not finding the same things funny and not really into the whole partying…it’s almost like I just skipped a couple years. I had to kinda take control of my life because it was either lay down and die or get up and fight kind of thing. And obviously no one else can do that for you so I kind of had to learn my disease and I was getting diagnosed with little minor things so I kind of had to like take a grip on all my meds, everything. (19-year-old female with JIA)

I had to mature a little faster [than my friends]…I mean, [Crohn’s is] kind of an adult thing to have to deal with. I would say all of the stuff in the maturation process like driving or stuff like that has all been the same as my friends. I would say just as far as dealing with it can make me a

little more responsible earlier than some other people might have developed a similar sense of responsibility. (19-year-old female with IBD)

When Crohn's first hit me, it was kind of during a time when I was becoming a teen, so I didn't really mature as much then. But once I wasn't affected by the Crohn's so much, then I kind of went through it faster… when I'm not feeling well or I'm stuck in the hospital, I'm not really going to get a chance to change my outlook on life or whatever. But then after all the experiences, when I got through it, that kind of made my personal view different. (17-year-old male with Crohn’s)

I believe everyone started drinking long before I started drinking. I didn't start drinking until I was like 16 or 17.…I always thought about how [drinking] would affect my Crohn’s. I’ve always heard about like alcohol can disturb the stomach lining. Therefore I was like, well, if my stomach’s already affected, why add poison to it? (19-year-old male with IBD)

Like I kind of just have to pay like more attention to what I do during my daily life. And I've had to like adjust my life according to what aggravates the arthritis and what doesn't. I think because [managing my arthritis] consumes so much of my life that I wouldn’t want to just like throw it away in one night [of drinking]. (19-year-old female with JIA)

I know if you black out and don't know how many you've had you're going to wake up with your blood sugar all out of whack. If you drink way too much and you don’t give yourself enough insulin, you wake up and your blood sugar is way too high and you feel like crap. That's just common sense though, I don't black out. But I know like if you don't give yourself enough insulin, your blood sugar is going to go up. And if you black out and don't know how much you drank, your blood sugar is really going to go up you know. (17-year-old male with T1D)

When you are drunk…God forbid if I get sick enough that I’m like throwing up, that’s like always scary because you never know what’s going to trigger this disease because there isn’t concrete stuff on it in terms of food or whatever… I drank for the first time when I was 16. It was once and I didn't drink again until I was 17 and then I only drank a couple times that year. So it wasn't until my senior year of high school when I was 18 that I actually like drank every once and a while. (19-year-old female with IBD)

I know I can't really skip [my medication] because then my knees are going to hurt really bad. So I guess if I ever drink in the future, I'll just barely have any because I'm afraid that it's going to like cancel it out or something… When I woke up that morning it was really bad. I don't know if it was because I didn't take my medicine or because actually drinking. (16-year-old female with JIA)

When I was diagnosed like I felt really sick, like couldn't walk or anything. If I do [drink alcohol] and the medicines stop working, I don't want to go back to where I was. I'd rather just stick with the medicine and have it work. I read like before my bottles like of medicine. It's like,

"Do not take with alcohol." And I just like went online, if it's like good to drink alcohol with medicine, and basically it just said like, "No, don't because it won't work, like the medicine." (16-year-old female with polymyositis)

Dr. X basically told me "if you drink you will die." Like, I'm not exaggerating or anything he completely said, "drinking is fatal for someone with arthritis on your medication." And, like, he's a great doctor and I've had a great experience with him but I mean, to say that completely freaked me out going to college. This was right before college so I was nervous about it and it definitely has been hard because I can't like do whatever I want. Other people can just get black-out drunk and they're fine the next day, but I know I could absolutely never do that [because of my medication]. And it's hard because you do those kind of things to like, relieve stress, but those kind of things actually stress me out more because I know that like, if I'm doing it, it's always risky [because I'm taking methotrexate]. But, I have a different doctor who's more of a like a nutritionist holistic person and he's like, an insanely smart person and he basically told me that what Dr. Y said is kind of false because with the amount of methotrexate I'm on and the amount of time it takes to leave your system, like it really wouldn't affect me. (18-year-old female with JIA)

If one day I'm planning on going out with my friends and my stomach hurts…I won't drink…I always listen to my body. I think your body will tell you what you need. You know, if you are hungry your body will tell you. You know if you are thirsty your body will tell you. Like your body will tell you what it needs and what's okay and what's not okay. (19-year-old female with IBD)

Well I [decided not to drink that night because I] was just listening to my body, I guess. I didn't feel great. You know, when my blood sugar's pretty high like that I just don't feel good at all. I feel like, dehydrated, and I just feel kind of sick. So you know once I start feeling like that, I really try to focus first on getting my blood sugar in check so I can feel better first and then go back to whatever I was doing. I mean for the most part I've been pretty good about keeping it in line and getting my blood sugar where it needs to be if I'm drinking or anything like that so, I mean it's not often, but I mean, I don't know- a few times over the school year, I'd say. I'm not like a crazy drinker or anything so only a few times I'll skip out. (19-year-old male with T1D)

It's just all about feeling if you're high or low. And you can lose that if you get too drunk. I definitely try to pace myself like don't drink a lot too fast. And I'll try to limit a little bit. Like if I know I've had a lot to drink, I'll be like "okay I'm done." Even if my friends keep drinking. (18-year-old male with T1D)

When I don't feel right, I feel like something is wrong. So I go check my blood sugar, which is the first thing I do just in case it is something with my diabetes. But if it is not with my diabetes, then I look it up in the computer, like what are these things for? And if things don't get better, I talk with my mom and then we make an appointment…So when people drink alcohol, I don't

think they are going to pay attention to their symptoms. Because the brain is not in place to pay attention to what they are doing...I like to have the knowledge of what I'm doing. (17-year-old male with T1D)

[When I smoked marijuana] I thought I couldn't breathe. [My friends] weren't sure what to do because they weren't sure if it was just because I had you know, had [marijuana] in my system, or like was it because I'm actually hurt and slowly not being able to breathe…it was very confusing. They didn't know what to do. And I was like, "It's okay, it's okay. I'll be fine. Just give me lots of water. I felt...gross. There was nothing -- I couldn't breathe so it would get hot and I felt like I was being funny. I was being funny and I wasn't sure what to do because I wasn't even sure myself if it was actually happening or not. So it was confusing...and it was a very horrible experience. (19-year-old female with asthma)

When you are drunk, you don't feel things as strongly as like other times...because those feelings are kind of lessened, I'm not as able to be like, "well this is feeling…" you know something like that. (19-year-old female with IBD)

Yeah, [I've given] a lot of thought [to how my body might react when drinking], actually 'cause like you can't trust other people to know what's happening to your body so you have to be able to tell for yourself. I would check my sugar [when I was drinking] and I realized that I had eaten a lot so it was on the higher side. So I'm sure 'cause the alcohol wanted me to eat, so that's what I did but it didn't go well at all. And I went high, it went to like 270 or so, I think something like that, I don't remember. (17-year-old female with T1D)

In my early teens, I discovered that, okay, even though marijuana makes me feel really funny in the head and kind of makes me want to go to sleep, [Marijuana] like *killed* my Crohn's pain… for the longest time, and it's like, yeah, I can do this for recreational because yeah because I know it's not gonna kill me. But primarily, that's what I used it for -- pretty much as a really good painkiller. I noticed that out of all the pills I did, me smoking marijuana in a five-year span, I've had far less hospitalizations, far less attacks, so I pretty much just stuck with it. (19-year-old male with IBD)

…I smoked because it would make my knees stop hurting or help me sleep because I usually can't sleep at night. Whenever I smoke my knees don't hurt for like a while. Even if I didn't take like Advil or something. (16-year-old-female with JIA)

I really enjoy drinking just because I do feel, like, relaxed when I do it and when you have arthritis, like everything is so tight…and when you drink it's just sort of like you're more loose. And I mean, I don't know if drugs are a topic in this too, but definitely smoking for me too has definitely been [something I've done]. I haven't done it too much because I've been nervous about the arthritis- but it's been amazing in terms of like, how it makes you feel. I've smoked marijuana, but I would never do like hard drugs because I mean people who have normal bodies flip out from that so that's why I never would do it. (18-year-old female with JIA)

It honestly just calms it down. I've done like my own little 'studies' I call them. If I know I'm feeling like my stomach's like upset and I'm having like loose stool and whatnot it’s like, oh, well maybe if I try to smoke maybe this will help. It's a very calming effect on my stomach. I know I'm eligible to get a medical marijuana card, but I don't know. I feel like it's just a judgy thing, you know what I mean? (19-year-old female with IBD-associated arthritis)
